# Supplementary material for: A gene regulatory network to control EMT programs in development and disease
Source: Nat Commun. 2019 Nov 11;10:5115. doi: 10.1038/s41467-019-13091-8 (PMC6848104; doi:10.1038/s41467-019-13091-8)
Supplement: Supplementary file 1 — Supplementary Material [file 41467_2019_13091_MOESM1_ESM.pdf]

Supplementary material

A Gene Regulatory Network to Control EMT Programs in Development and Disease

Fazilaty et al.

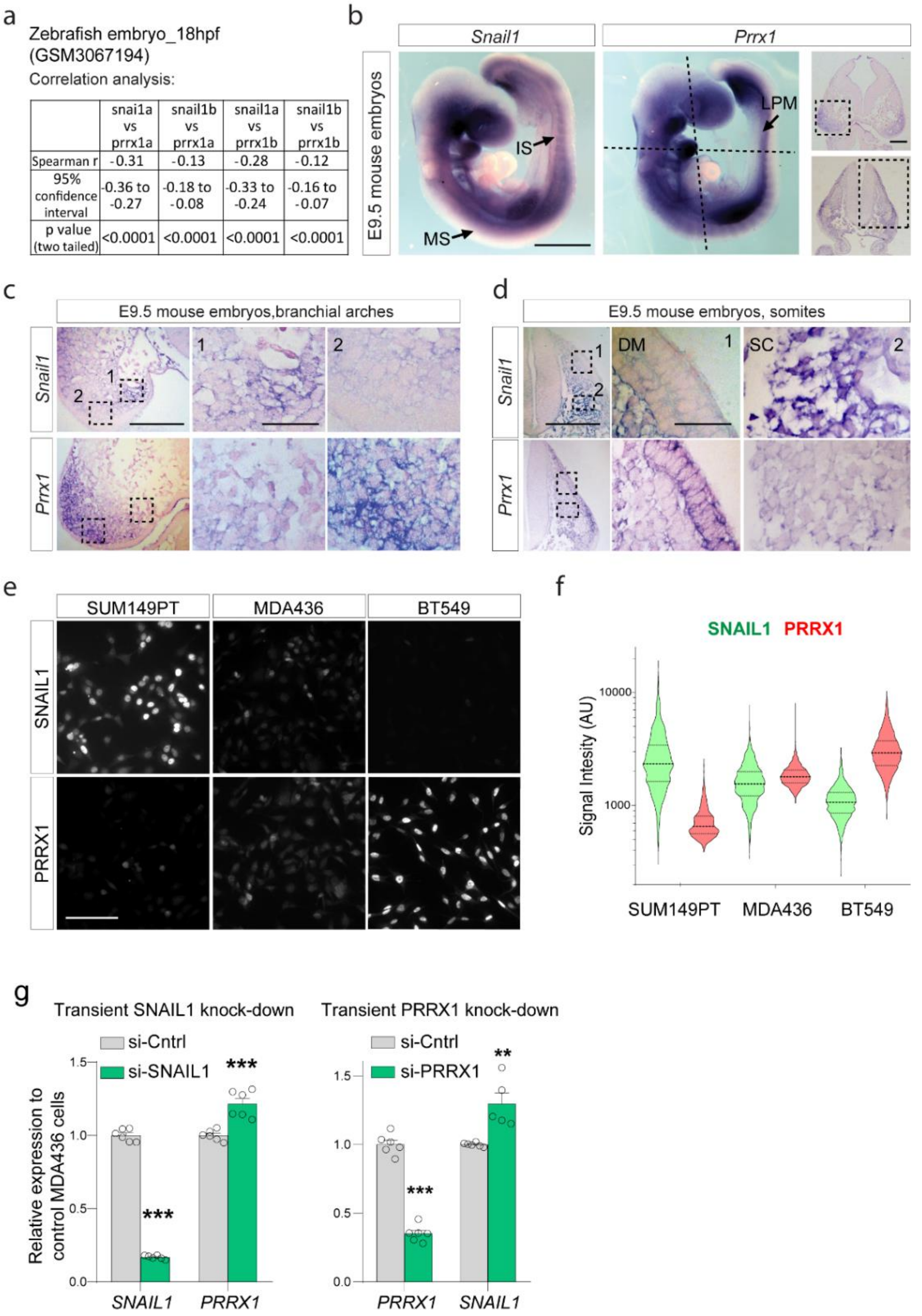

Supplementary Figure 1. Snail1 and Prrx1 complementary expression in development and disease. (a) Correlation analyses between each pair of *snai1*a/b and *prrx1a/b* genes extracted from scRNA-seq data (GSM3067194) of 18hpf zebrafish embryos. (b) Expression of *Snail1* and *Prrx1* in lateral views of E9.5 mouse embryos. Scale bars: 250 μm for whole mounts and 100 μm for sections. (c-d) Transverse sections of the embryos shown in (b) to better observe the branchial arches (c) and the somites (d), together with higher power pictures of the boxed areas (1-2). Complementary expression is observed in the branchial arches and the somites. Scale bars: 100 μm for sections and 25 μm for insets (boxes 1-2). (e) Double IF for SNAIL1 and PRRX1 in three breast cancer cell lines, SUM149PT, MDA436 and BT549, with different levels for each protein. Scale bar: 100 μm. (f) Signal intensity analyses for SNAIL1 and PRRX1 in the three cell lines show a gradual reduction of SNAIL1 concomitant with a gradual increase for PRRX1 levels from SUM to BT cells. (g) qPCR assay showing upregulation of *PRRX1* (left) and *SNAIL1* (right) transcription upon transient knock-down of SNAIL1 or PRRX1, respectively, in MDA436 cells. hpf: hours post fertilization; IS: immature somites; MS: mature somites; LMP: lateral plate mesoderm; DM: dermomyotome; SC: sclerotome; AU: arbitrary units; si: short interfering RNA (siRNA). Bars represent mean plus SEM, n=6 independent experiments as biological replicates and asterisks indicate significant p-value in t-test (** $p < 0.01$ and *** $p < 0.001$). Source data are provided as a Source Data file.

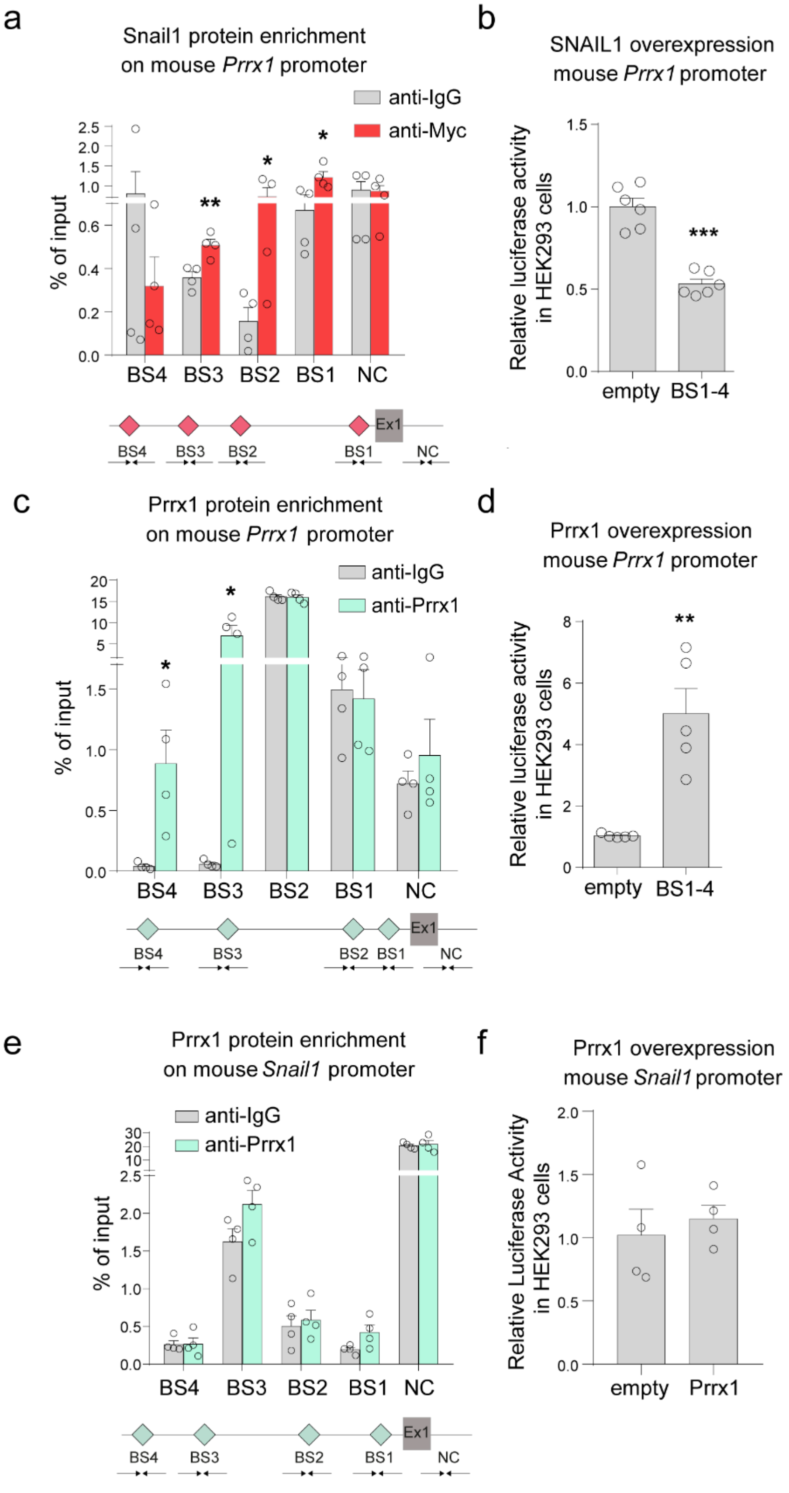

Supplementary Figure 2. Snail1 and Prrx1 act in an antagonistic manner on *Prrx1* promoter in the mouse. (a) Snail1 enrichment in the mouse *Prrx1* promoter shown by ChIP assay in NIH3T3 cells using anti Myc antibody (for Snail1-Myc overexpression). A schematic map is shown, where red diamonds represent Snail1 potential binding sites (E-boxes; CANNTG) on *Prrx1* promoter. (BS1: -265, BS2: -2100, BS3: -3028, BS4: -4074 and NC: +2099) Ex1: Prrx1 exon 1 (n=4). (b) Decrease in mouse *Prrx1* promoter activity after SNAIL1 transfection in HEK293 cells (n=6). (c) Prrx1 directly binds to its own promoter, as assessed by ChIP assays in NIH3T3 cells using Prrx1 specific antibody. A schematic map is shown, where cyan diamonds represent Prrx1 potential binding sites (TAATKDS) on its own promoter. (BS1: -114, BS2: -662, BS3: -2113, BS4: -4053 and NC: +2099) Ex1: Prrx1 exon 1 (n=4). (d) Activation of mouse *Prrx1* promoter after Prrx1 overexpression in luciferase assays in HEK293 cells (n=5). (e) Lack of enrichment for Prrx1 binding to mouse *Snail1* promoter in ChIP assays in NIH3T3 cells with a Prrx1 antibody. (BS1-4: -3110, -2827, -2667, -1714 and NC: +302). Cyan diamonds represent Prrx1 potential binding sites on *Snail1* promoter (n=4). (f) Luciferase assays in HEK293 cells showing the lack of significant difference in the activity of mouse *Snail1* promoter after Prrx1 overexpression. Arrows represent primers used for qPCR amplification. Locations of red and cyan diamonds represent distances between BSs and the promoter. Ex1: *Snail1* exon 1 (n=4). BS: biding site and NC: negative control region, which does not contain potential BS. Symbols in binding sites are as follows. K: T/G, D: G/A/T, S: G/C and N: G/A/T/C. Bars represent mean plus standard error of the mean (SEM), indicated (n) represent number of independent experiments as biological replicates and asterisks indicate significant p-value in t-test (* $p < 0.05$, ** $p < 0.01$ and *** $p < 0.001$). Source data are provided as a Source Data file.

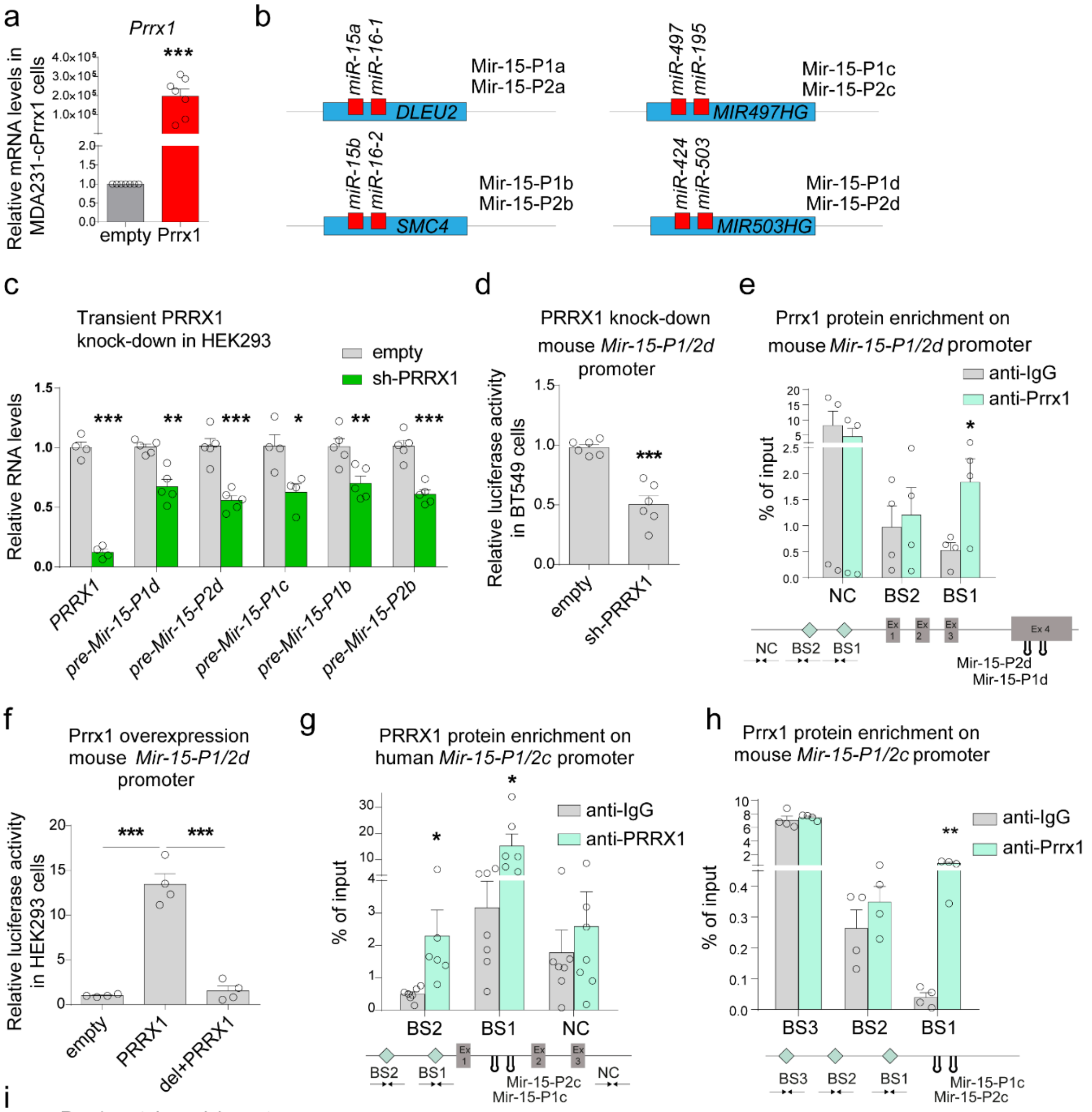

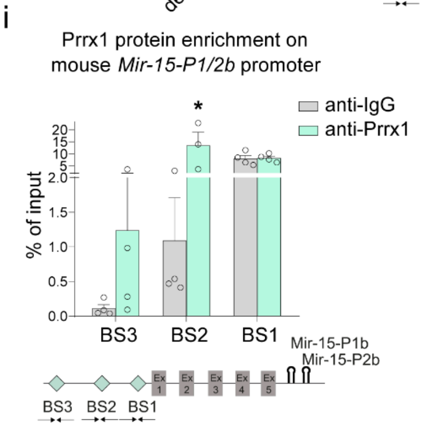

Supplementary Figure 3. PRRX1 directly induces the transcription of *miR-15* family members. (a) *Prrx1* upregulation upon conditional (Dox-mediated) Prrx1 overexpression by qPCR in MDA231 cells (n=7). (b) Schematic representation of miR-15 family members in the human genome and their updated nomenclature[25]. (c) qPCR assay showing downregulation of *PRRX1* and *premiRNAs* transcription upon transient knock-down of PRRX1 in HEK293 cells (n=4). (d) Repression of human *Mir-15-P1/2d* promoter upon knock-down of PRRX1 by specific shRNA shown by dual luciferase assay in BT549 cells (n=6). (e) Prrx1 directly binds to the mouse *Mir-15-P1/2d* promoter shown by ChIP assay in NIH3T3 cells using Prrx1 specific antibody. (BS1: -2069, BS2:-2972 and NC:-4512) (n=4). (f) Activation of mouse *Mir-15-P1/2d* promoter after Prrx1 transfection in HEK293 cells. This activation is abolished upon deletion of the Prrx1 binding site in *Mir-15-P1/2d* promoter (n=4). (g-i) Prrx1 directly binds to the human *Mir-15-P1/2c* (BS1: -214, BS2: -2134 and NC: +5141) (n=6), mouse *Mir-15-P1/2c* (BS1:-627) (n=4) and *Mir-15-P1/2b* (BS1: -999 and BS2: -1386) (n=4) promoters shown by ChIP assay in BT549 and NIH3T3 cells, respectively, using Prrx1 specific antibody. Locations of cyan diamonds represent distances between BSs and the promoter. cPrrx1: conditional Prrx1 overexpression; BS: biding site and NC: negative control region, which does not contain potential BS. Bars represent mean plus standard error of the mean (SEM), indicated (n) represent number of independent experiments as biological replicates and asterisks indicate significant p-value in t-test for all except f for which one-way ANOVA with Bonferroni's multiple comparison test is performed (* $p < 0.05$, ** $p < 0.01$ and *** $p < 0.001$). Source data are provided as a Source Data file.

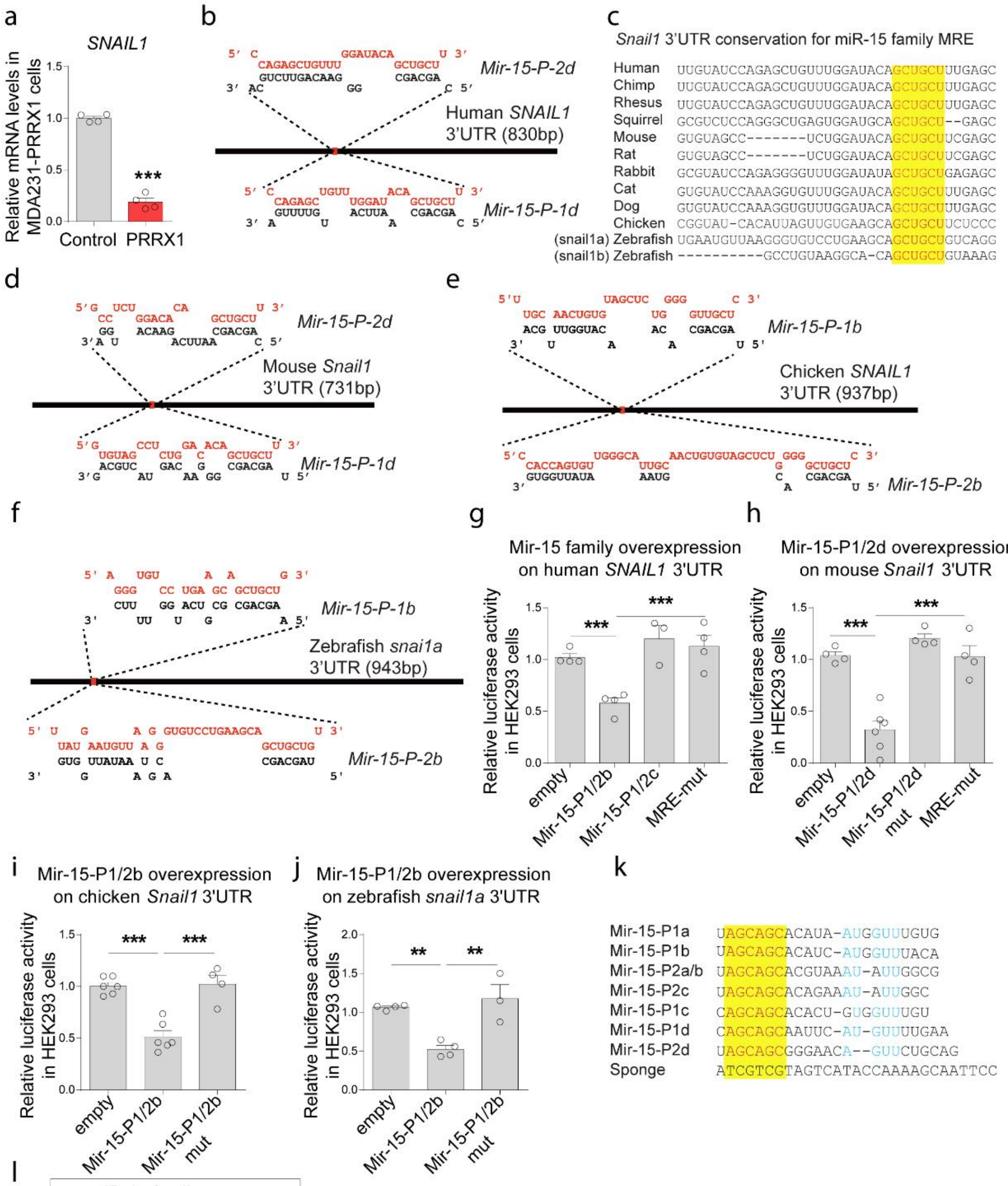

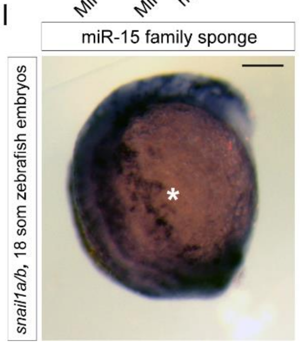

Supplementary Figure 4. Repression of Snail1 by *miR-15* family is conserved. (a) qPCR assays after stable overexpression of PRRX1 confirms the observed downregulation of *SNAIL1* in microarrays (n=4). (b) Schematic representation of human *SNAIL1* 3'UTR, predicted to be a target of *Mir-15-P1/2d*, by RNAhybrid (https://bibiserv2.cebitec.uni-bielefeld.de/rnahybrid). (c) Conservation of miR-15 family binding sites in *SNAIL1* 3' UTR sequences in vertebrates. MRE is highlighted. (d-f) Schematic representation of mouse, chicken and zebrafish *Snai1* 3'UTRs, predicted to be targets of *Mir-15* family members of each species. (g-j) Luciferase assays in HEK293 cells showing. (g) repression of human *SNAIL1* 3'UTR by *miR-151/2b* family members. This repression is abolished upon mutation of *MRE* in *SNAIL1* 3'UTR (n=4). (h-j) Similar experiments to those shown in (d), but using either mouse or chicken *Snail1* or zebrafish *snail1a* 3'UTR regions, respectively. These repressions are abolished upon mutations in the miRNAs seed sequence (n=4 except for i that n=6). (k) Sequence alignment for members of the miR-15 family, and the partially complementary sequence of the Sponge used. The seed sequence is highlighted in yellow box and blue nucleotides indicate additional conserved residues within family members. MRE: miRNA responsive element. (l) miR-15 family sponge-injected zebrafish embryos showing *snail1a/b* expression by *in situ* hybridization. Note the ectopic *snail1* positive cells that migrated beyond their normal position shown by asterisk. This very extreme phenotype is observed in a small percentage of embryos. Scale bar: 250 μm. Bars represent mean plus standard error of the mean (SEM), indicated (n) represent number of independent experiments as biological replicates and asterisks indicate significant p-value in t-test for a and one-way ANOVA with Bonferroni's multiple comparison test for the rest (* $p < 0.05$, ** $p < 0.01$ and *** $p < 0.001$). Source data are provided as a Source Data file.

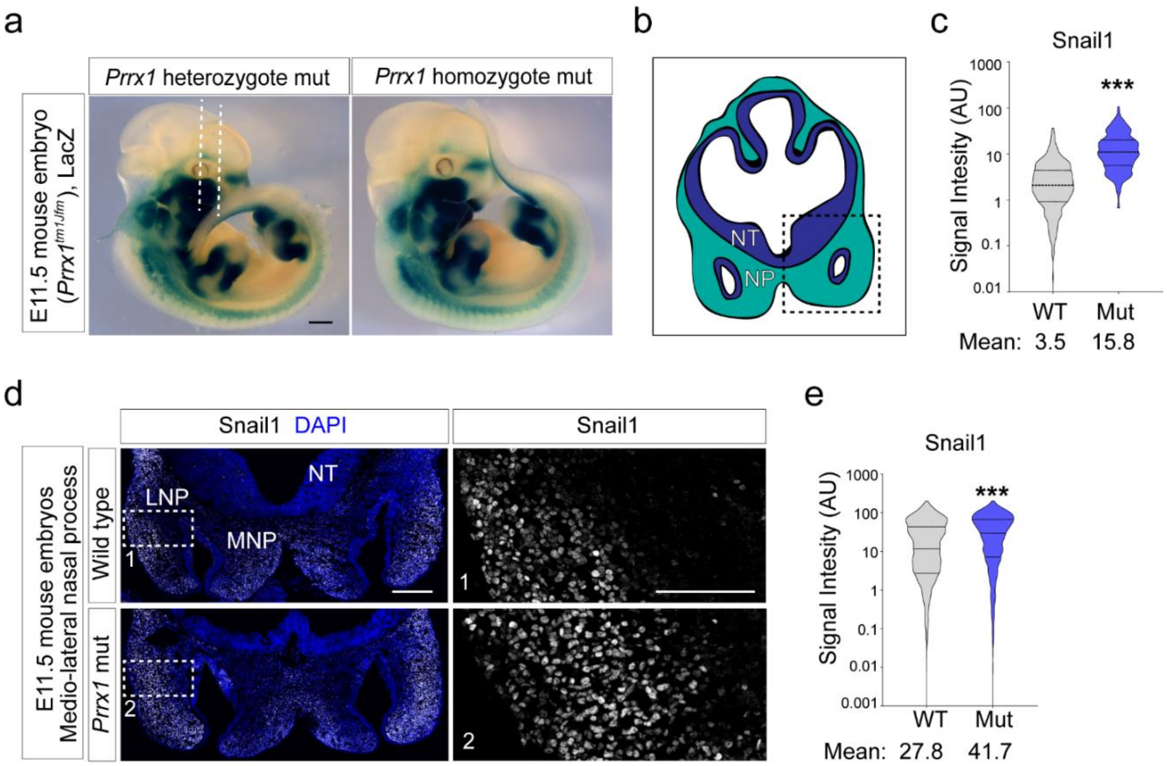

Supplementary Figure 5. Repression of Snail1 through Prrx1-induced miR-15 family in the mouse. (a) LacZ staining of E11.5 hetero or homozygous *Prrx1* mutant embryos, (129*S-Prrx1tm1Jfm)*[1]*.* Scale bar: 1mm*.* (b) Schematic representation of transverse sections of cranial regions of E11.5 embryos at the level of the nasal pit. (c) Signal intensity for Snail1 is measured by calculating the average signal in the nuclei of all cells in the selected regions of nasal pit of Snail1 IF in Figure 4i. (d) IF in the nasal process region of E11.5 WT and mutant embryos show increased levels as well as expansion of Snail1 expression domains. Scale bar: 250 μm for sections and 100 μm for insets (boxes 1 and 2). (e) Signal intensity for Snail1 is measured calculating the average signal in the nuclei of all cells in the selected regions of lateral nasal process in d. WT: wild type; Mut: mutant; NT: neural tube, NP: nasal pit; MNP: medial nasal process, LNP: lateral nasal process; AU: arbitrary units. Asterisks indicate significant p-value in t-test (* $p < 0.05$ and *** $p < 0.001$). Quantifications are performed for one section of WT or mutant embryos, and the increase and expansion is observed in n=2/2 mutant embryos compared to n=3/3 different E11.5 WT. Source data are provided as a Source Data file.

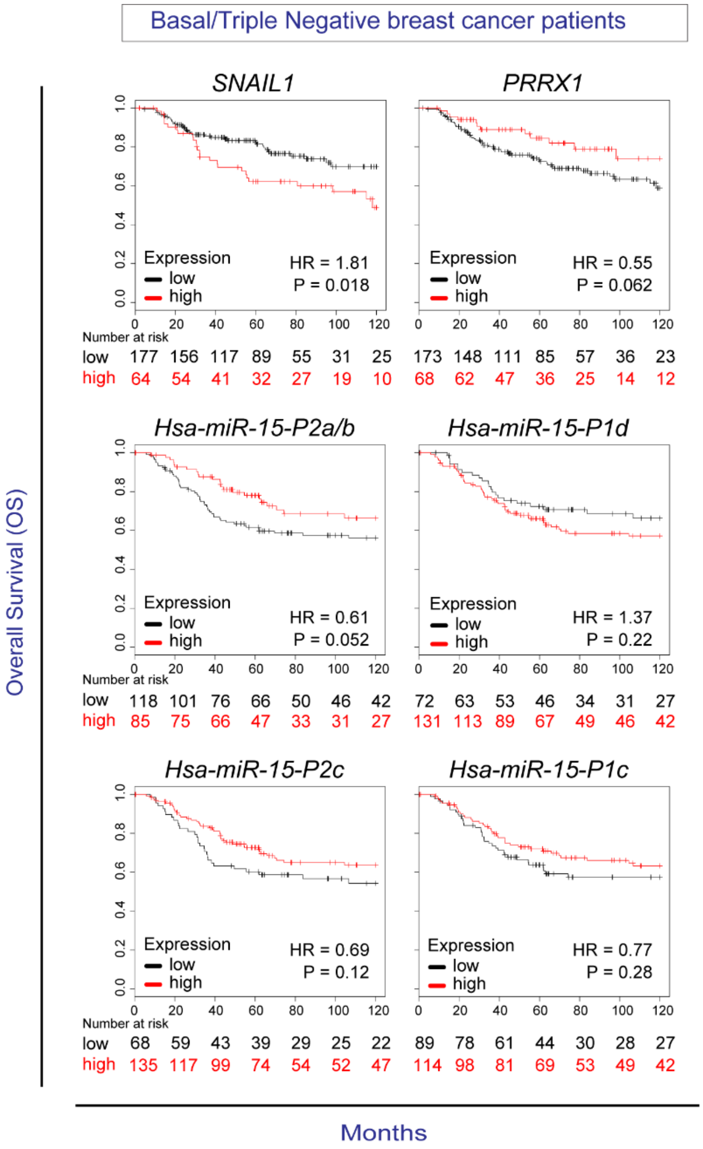

Supplementary Figure 6. Expression of miR-15 family members correlates with Prrx1 in survival of triple-negative breast cancer patients. Kaplan-Meier overall survival (OS) plots from basal/triple-negative breast cancer patients showing that high expression of *SNAIL1* correlates with low survival, while *PRRX1* high expression correlates with a better survival. Expression of *miR-15* family members follow a similar trend as that of *PRRX1*. Hazard ratio (HR) and logarithmic ranked p Value (longrank P) were analyzed to infer the significance of the differences. Numbers below each graph represent number of patients at risk in any given time (months), black for low expression and red for high expression of each gene/miRNA. The cut-off is automatically calculated based on the best performing threshold.

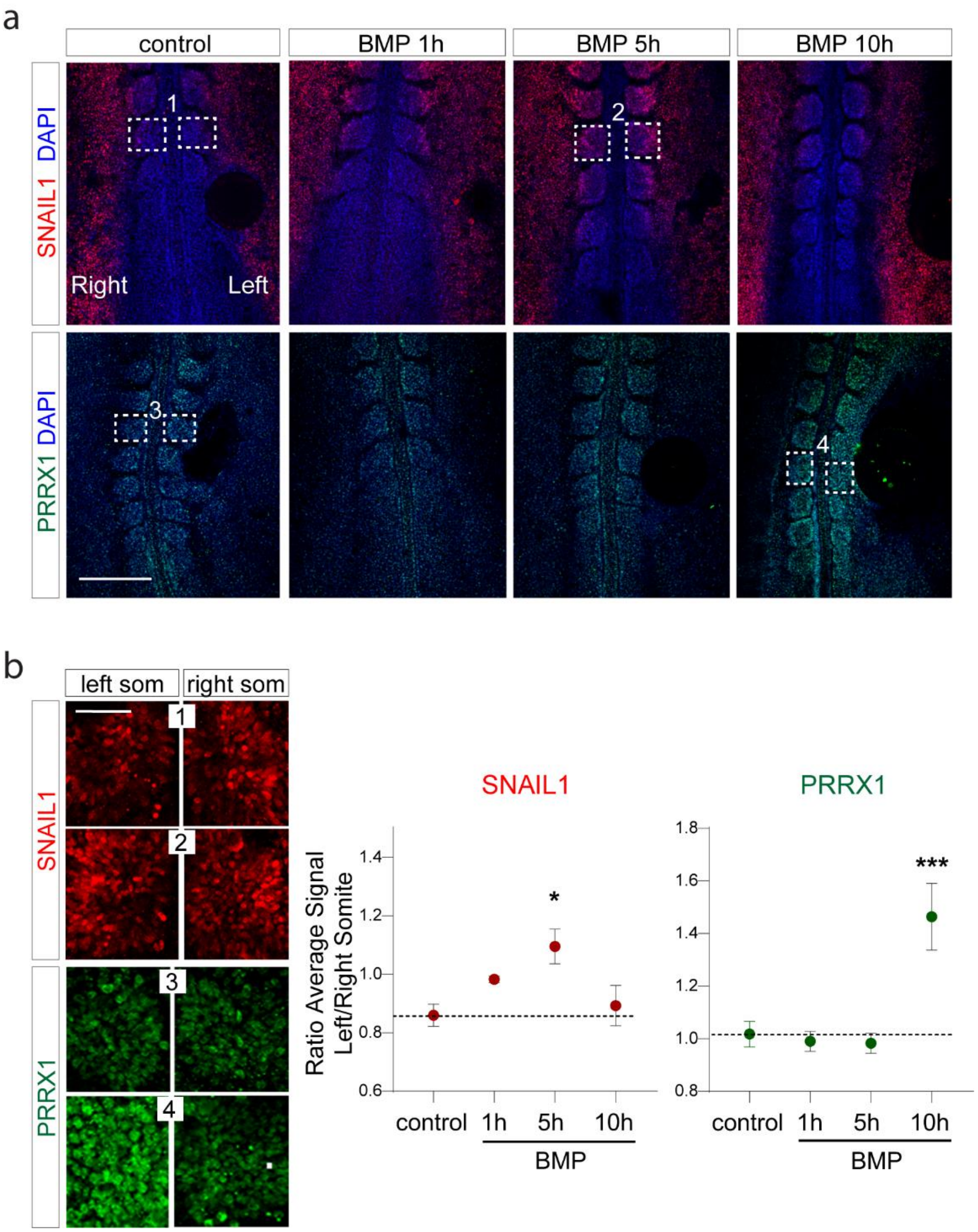

Supplementary Figure 7. Sequential upregulation of Snail1 and Prrx1 upon BMP treatment in zebrafish and chicken embryos. (a) Ventral view of chicken embryos showing SNAIL1 or PRRX1 protein expression by IF in control embryos and at different times after BMP-soaked bead implantation. Insets are shown at the levels of left and right somites in the vicinity of bead. Scale bar: 100 μm. (b) Signal intensity for SNAIL1 or PRRX1 protein expressions are measured by calculating the average signal in 3/4 somites in each side of each embryo, and the average ratio of left/right is represented for each time point. Numbers indicate the region of the inset in a, shown as dashed boxes. Total of n=6-8 somites from n=2 embryos were quantified for each condition at each time point. The value from each somite is considered as separate for the statistical test. Scale bar: 50 μm. Som: somite. Asterisks indicate significant p-value in One-way ANOVA with Bonferroni's multiple comparison test compared to the control (* $p < 0.05$ and *** $p < 0.001$). Source data are provided as a Source Data file.

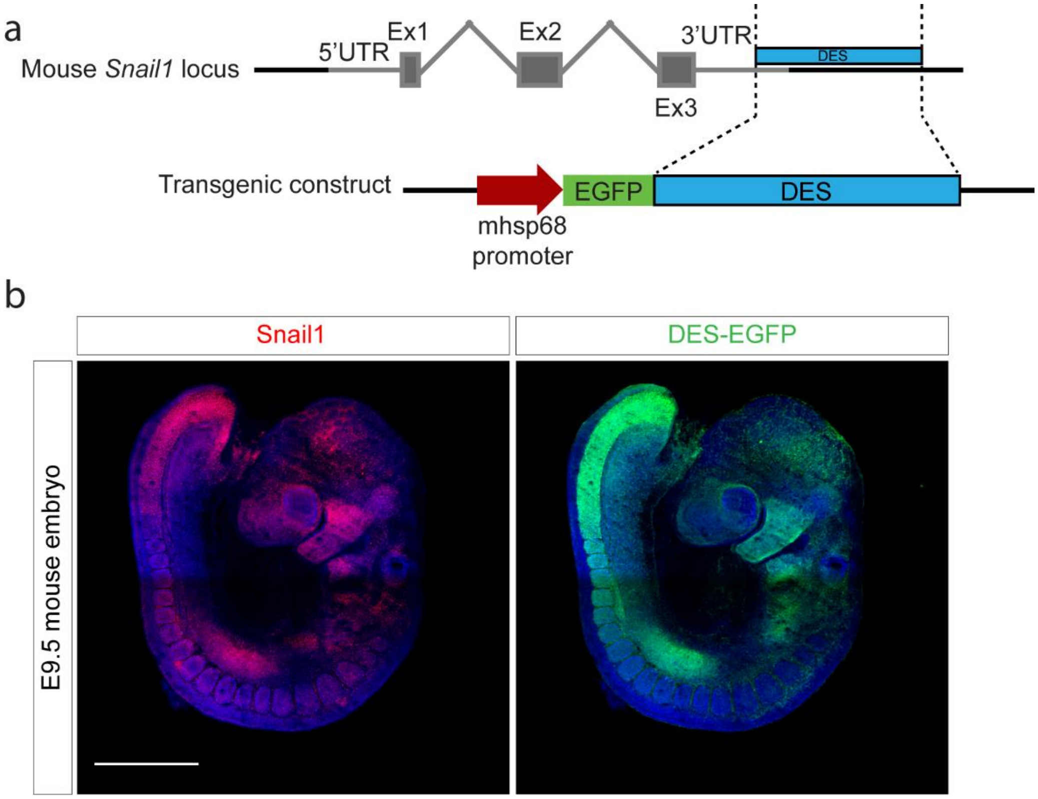

Supplementary Figure 8. DES-GFP transgenic mouse recapitulated Snail1 endogenous expression. (a) Representation of the construct used to generate the transgenic DES-EGFP Snail1 line and the localization of the DES enhancer in the mouse *Snail1* locus. (b) Double IF for Snail1 and GFP in E9.5 transgenic mouse embryo showing endogenous Snail1 and EGFP expression corresponding to Snail1. Scale bar: 250 μm. UTR: untranslated region; Ex: exon; DES: downstream enhancer of Snail1; EGFP: enhanced green fluorescent protein; mhsp68 promoter: mouse heatshock protein 68 minimal promoter.

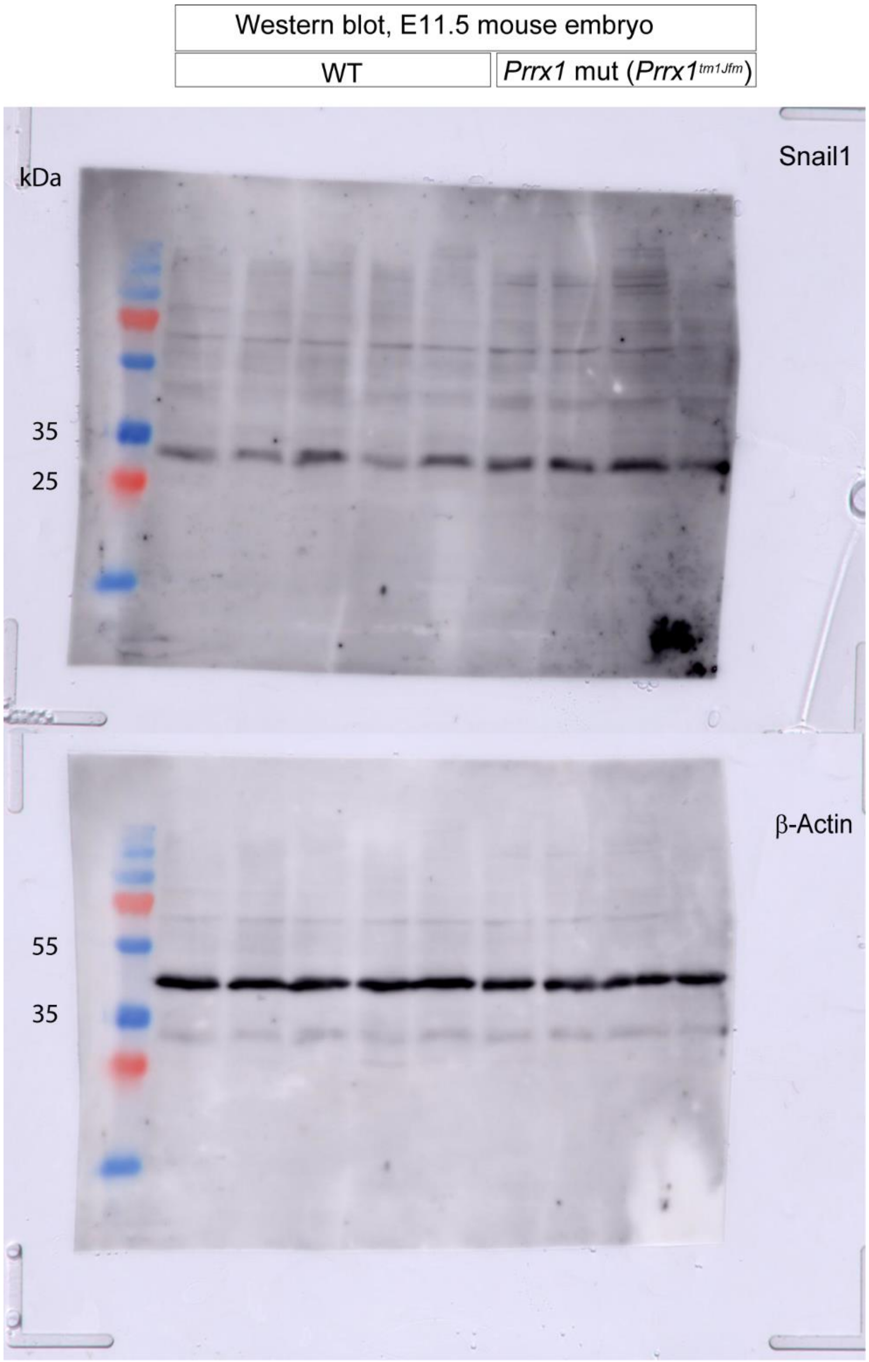

Supplementary Figure 9. Uncropped and unprocessed western blot for Snail1 and β-actin from E11.5 control and Prrx1 mutant embryos.

Supplementary Table 1, GEO datasets									
GEO acc. #	Sample scRNA-seq	Total cell #	# of cells	# of Snail1 single + cells	# of Prrx1 single + cells	# of cells Snail1 high and Prrx1 low/-	# of cells Snail1 low/- and Prrx1 high	# of cells Snail1 high and Prrx1 high	Ref.
GSE87038	E9.5 mouse embryos	764	440	219 (49%)	87 (19%)	245 (55%)	143 (32%)	54 (12%)	[2]
GSE103322	18 head and neck cancer patients	5902	1718	480 (28%)	1005 (58%)	564 (32%)	1031 (60%)	123 (7%)	[3]
GSE75688	11 breast cancer patients	551	138	96 (69%)	22 (16%)	103 (74%)	28 (20%)	7 (5%)	[4]
GSM3067194	18hpf zebrafish embryos	6962	1620	snail1a: 582 (36%)<br><br>snail1b: 98 (6%)	prrx1a: 376 (23%)<br><br>prrx1b: 171 (10%)	snail1a: 787 (48%)<br><br>snail1b: 116 (7%)	prrx1a: 627 (38%)<br><br>prrx1b: 332 (20%)	prrx1a: 52 (1.6%)<br><br>prrx1b: 2 (0.1%)	[5]

Supplementary Table2, miR-15 family nomenclature and accession numbers	
miR-15 family	MIPF0000006
Hsa-Mir-15-P1d (hsa-miR-424)	MI0001446
Hsa-Mir-15-P2d (hsa-miR-503)	MI0003188
Hsa-Mir-15-P1b (hsa-miR-15b)	MI0000438
Hsa-Mir-15-P2b (hsa-miR-16)	MI0000115
Hsa-Mir-15-P2c (hsa-miR-195)	MI0000489
Hsa-Mir-15-P1c (hsa-miR-497)	MI0003138

Supplementary Table 3. Primer sequences	
Human miR424-503-promoter-F (Cloning)	GACTGCTAGCTTCAATATCATTCTCACAAATACAAAATAG
Human miR424-503-promoter-R (Cloning)	GATCCTCGAGTGGAACAACGTAGTGGGTGA
Human miR424-503-F (Cloning)	GACTGGATCCGGCTTCCTTCAGTCATCCAG
Human miR424-503-R (Cloning)	GACTCTCGAGTCTACCTGAGCAGGGAAAGG
h-424-mut-F	GATCCCCCTTCATTGACTCCGAGGGGATACTATTATAATTCATGTTTTGAAGTGTTCTAAATGGTTC
h-424-mut-R	GAACCATTTAGAACACTTCAAAACATGAATTATAATAGTATCCCCTCGGAGTCAATGAAGGGGGATC
h-503-mut-F	GTGCCCGCGCTCAGCCGTGCCCTTATTATGGGAACAGTTCTGCAGTGA
h-503-mut-R	TCACTGCAGAACTGTTCCCATAATAAGGGCACGGCTGAGCGCGGGCAC
miR-15b-16-2-cloning-F	GACTGGATCCACTAAAGCTTGAAAGAGTGTTCTTCTGT
miR-15b-16-2-cloning-R	GACTCTCGAGATAAACAAAAGGGACAGATTATCAAAAG
QPCR HsPrrx1 FW	CTGATGCTTTTGTGCGAGAA
QPCR HsPrrx1 RV	ACTTGGCTCTTCGGTTCTGA
hSNAI1-F	GCTGCAGGACTCTAATCCAGA
hSNAI1-R	ATCTCCGGAGGTGGGATG
QPCR HsPrrx1 FW	CTGATGCTTTTGTGCGAGAA
QPCR HsPrrx1 RV	ACTTGGCTCTTCGGTTCTGA
premir424-F	TTGACTCCGAGGGGATACAG
premir424-R	GACCCCACCTTCTACCTTCC
premir503-F	GCGAGTCGAGGAGAGACG
premir503-R	GAACGGCAGTCCCAGACTTA
hsa-TBP-F	CGGCTGTTTAACTTCGCTTC
hsa-TBP-R	CACACGCCAAGAAACAGTGA
h-PRRX1-del1-F	TTATTCGTCTACCTTCAGAAGATTCTCTTCCACACTAATTCG
h-PRRX1-del1-F	CGAATTAGTGTGGAAGAGAATCTTCTGAAGGTAGACGAATAA
h-PRRX1-del2-F	TTCAGAGTGGGTTTTTTTTTTAATGCAATATTGTATTCAAACAAAAAGAGGG
h-PRRX1-del2-F	CCCTCTTTTTGTTTGAATACAATATTGCATTAAAAAAAAAACCCACTCTGAA
h-PRRX1-del3-F	GGAGAAAGTTGTGGAGTTTTACATGAACATTTGCTAAACATGTTTT
h-PRRX1-del3-F	AAAACATGTTTAGCAAATGTTCATGTAAAACTCCACAACTTTCTCC
miR-15b-16-2-cloning-F	GACTGGATCCACTAAAGCTTGAAAGAGTGTTCTTCTGT
miR-15b-16-2-cloning-R	GACTCTCGAGATAAACAAAAGGGACAGATTATCAAAAG
mmu-miR,15bmut-F	GCTGAGTCCTGTCTTTTGGAACCTTAAAGTACTGTTCGTCGACATCATGGTTTACATACTACAG

mmu-miR,15bmut-R	CTGTAGTATGTAAACCATGATGTCGACGAACAGTACTTTAAGGTTCCAAAAGACAGGACTCAGC
mmu-miR,16mut-F	GTATTATGTTTGGATATCTGACATGCTTGTTCCACTCTTCGTCGACGTAAATATTGGCGTAGTGAAATAAAT
mmu-miR,16mut-R	ATTTATTTCACTACGCCAATATTTACGTCGACGAAGAGTGGAACAAGCATGTCAGATATCCAAACATAATAC
m497-PxB-F	CTAAAAACAGGCCTTATTGACTACA
m497-PxB-R	GATGTGGCTTTACTTTGTGAAGATT
m497-up-F	AGGATATTAGAATTGGGGAGGTTAGT
m497-up-R	TGAGTCCAATACATTAGAAAGAGGAGT
m497-dw-F	ACTTGCTCGTACAGGTTGTATATGTTCT
m497-dw-R	CTTGGGAAGACTAATAGGAGTTAAAGTG
miR-195mut-F	GTTGCCCACACCCAACTCTCCTGGCTCTTCGTCGACAGAAATATTGGCATGG
miR-195mut-R	CCATGCCAATATTTCTGTCGACGAAGAGCCAGGAGAGTTGGGTGTGGGCAAC
miR-497mut-F	CAGTCCTGCCCCCGCCCCTCGTCGACACTGTGGTTTGTACGG
miR-497mut-F	CCGTACAAACCACAGTGTCGACGAGGGGCGGGGGCAGGACTG
m195-497-clon-F2	GACTGGATCCCTAAACTACTTTTGCTGGTTCCTGATT
m195-497-clon-R2	GACTCTCGAGGACTTCTGTGTGATGGACATTTTTATAC
SNAI1,3',MRE503-F	TTTGTATCCAGAGCTGTTTGGATACACGACGATTGAGCTACAGGACAAAGGCTGACAG
SNAI1,3',MRE503-F	CTGTCAGCCTTTGTCCTGTAGCTCAATCGTCGTGTATCCAAACAGCTCTGGATACAAA
h503P-del1-F	AAAAAAAAACATGTTTAGCAAATGTATCATGTAAAACTCCACAACTTTCTCC
h503P-del1-R	GGAGAAAGTTGTGGAGTTTTACATGATACATTTGCTAAACATGTTTTTTTTT
h503P-del2-F	CCCTCTTTTTGTTTGAATACAATATTAGCATTAAAAAAAAAACCCACTCTGA
h503P-del2-R	TCAGAGTGGGTTTTTTTTTTAATGCTAATATTGTATTCAAACAAAAAGAGGG
hPRRX1-P-F2	ATTAGTAAATGTGTGCGGTGTTTTTCTTG
hPRRX1-P-R3	CTTTGTAGGGAATACAAGAAAATTGGAG
hPRRX1-P-R4	ATTAGTAAATGTGTGCGGTGTTTTTCTTG
497P-PXB1-F	AGGAGGGAGTGACTTCCAAAA
497P-PXB1-R	AAATTTGGGGTCCTCAGATACC
497P-PXB2-F	CTACCCAGATGTCTTTGGAGGT
497P-PXB2-R	CCGAAACAAAATATGAGGGTGT
497P-dw-F	TTATTGAGATACGGGACACAGC
497P-dw-R	CTCCAGCCCCTCCTCTATTTA
SMC4P-dw-F	AGCAAGACCCCATCTCTACAAA
SMC4P-dw-R	AGTGCACACGTAAAAGGACTGA
SMC4i-PXB1-2-F	TTGGATATGGTGGGAATCTTGT
SMC4i-PXB1-2-R	TCAATTGAACATGCACACAAAC
SMC4P-PXB3-F	GAACTTATTTCTTCCTGTGGGGTA
SMC4P-PXB3-R	CCTGGAAAAGACTGGGTTACAG
PRRX1P-dw-F	GGACTTACAGACATTCCCTTGG

PRRX1P-dw-R	GGCCTGAGAAACAAATAGATGG
PRRX1P-PXB1-F	CTCCCTTTCTCTCTAACTCTGATGTTG
PRRX1P-PXB1-R	GCCAACATCAGAGTTAGAGAGAAAG
PRRX1P-PXB2-F	TTGATTGCCTGCATTCTTACTT
PRRX1P-PXB2-R	TCAATGAGTCCAAAATGTAAACC
PRXP-B1-F2	GTACTTTTCTCATTGCCCTAGTCCT
PRXP-B1-R2	GTAAGCATATTAAGGCTATTTTTGGTTC
PRXP-B1-F3	AAGAAGGAGATTGTGATGGAGAAAG
PRXP-B1-R3	TTTTCCTTCTCAGTTGGATCAAGAA
PRXP-up-F	CCTAGCACCTGAGAAACATACTGATAAC
PRXP-up-R	ACTTAGCCTATAAATCAGAGAATACTTGT
PRXP-PXB3-4-F	AGACTATAACAGAGCAGATCAATTACCA
PRXP-PXB3-4-R	TCCCATATTTTAATGTTTTTAGGAGTCT
PRXP-PXB5-6-F	TTTATGAGGTGAACGCATTATCAG
PRXP-PXB5-6-R	TCTTGCTTGTTTAAGTCTTTGTGG
SNAI-B-1-2-R	CAGTTAGTAGTCCAGGTGTCTTGC
PRRX1P-F01Xho	GACTCTCGAGCCTTTATTCTCTTTCTTAATCCTCTCAA
PRX-B1-R2Nco	GACTCCATGGGTAAGCATATTAAGGCTATTTTTGGTTC
PRXP-CpG-R	GACTCCATGGAGGTTTCACCCTTAAAGGACATTC
PRXP-COM-R	GACTCCATGGAGGTGACTGACGGAGAAGTTCTTT
m503-F2	GACTGCTAGCCCCTCCCAAATTATTCCAAA
SNAI1-PXB3-5-F	CAACAGTTCTTTACTTTGCTTGGTT
SNAI1-PXB3-5-R	ACAGGGAAGGATTAAAACACCTAAG
SNAI1-PXB6-F	GACTTTGGCTTTTACTCTGAGACAG
SNAI1-PXB6-R	ACAGAGGCAGTAAGCAGTCATTAAG
SNAI1-PXB7-F	TCCCTTTGCATTGTAATTATCTGTT
SNAI1-PXB7-R	GTTCCTTCCCTTATCCAGTGTTTAC
SNAI1-dw-F	GCCCAAATTGTCAGTTTCATAAATA
SNAI1-dw-R	ACAAAGATGTAAACCAAGATCTCCA
EGFP_fwd	CCGACCTCTCTCCCCAGGGGGATCCGTGAGCAAGGGCGAGGAG
EGFP_rev	TAGTAGCTCCGCTTCCCTTGTACAGCTCGTCCATGC
PuroR_fwd	GCTGTACAAGGGAAGCGGAGCTACTAACTTCAGCCTGCTGAAGCAGGCTGG AGACGTGGAGGAGAACCCTGGACCTATGACCGAGTACAAGCCCACGG
PuroR_rev	GTCATTGGTCTTAAAGGTACCTCAGGCACCGGGCTTGCG
hUbC_promoter-F0	TCTTTCCAGAGAGCGGAACA
YFP-BamHI-F	TTAAGGATCCGCTCGTTTAGTGAACCGTCAG
YFP-KpnI+stop-R	TTAAGGTACCTACTTGTACAGCTCGTCCATGC
hPGK promoter-F	GTAGTGTGGGCCCTGTTCCT
3'LTR-R	TCGTTGGGAGTGAATTAGCC
hPRX1-enh-F	GACTGCTAGCGGTTTACTACTGGGGCAAGAGTT
hPRX1-enh-R	GACTCTCGAGAATATGGCAGAGAGGGTAAAAGG
hPRX1-enh-del-F	caaatatatatggtaggtgccacattatttgagagatgggtaag

hPRX1-enh-del-R	cttacccatctctcaaataatgtggcacctaccatatatatttg
hPRX-P-SN-BS4-del-F	cataatcagagtgtggatccaatctaattgctaatagaaaaaaaggaa
hPRX-P-SN-BS4-del-R	ttccttttttctattagcaattagattggatccacactctgattatg

References

1. Lu, M.F. *et al.* prx-1 functions cooperatively with another paired-related homeobox gene, prx-2, to maintain cell fates within the craniofacial mesenchyme. *Development* **126**, 495-504 (1999).
2. Dong, J. *et al.* Single-cell RNA-seq analysis unveils a prevalent epithelial/mesenchymal hybrid state during mouse organogenesis. *Genome biology* **19**, 31 (2018).
3. Puram, S.V. *et al.* Single-Cell Transcriptomic Analysis of Primary and Metastatic Tumor Ecosystems in Head and Neck Cancer. *Cell* **171**, 1611-1624 e24 (2017).
4. Chung, W. *et al.* Single-cell RNA-seq enables comprehensive tumour and immune cell profiling in primary breast cancer. *Nature communications* **8**, 15081 (2017).
5. Wagner, D.E. *et al.* Single-cell mapping of gene expression landscapes and lineage in the zebrafish embryo. *Science* (2018).
