# Supplementary material for: A gene regulatory network to control EMT programs in development and disease
Source: Nat Commun. 2019 Nov 11;10:5115. doi: 10.1038/s41467-019-13091-8 (PMC6848104; doi:10.1038/s41467-019-13091-8)
Supplement: Supplementary file 3 — Supplementary Dataset 1 [file 41467_2019_13091_MOESM3_ESM.pdf]

Supplementary Data 1.

Microarray data listing miRNA deregulation upon PRRX1 overexpression in MDA231 breast cancer cells

miRNA	MDA_C1	MDA_C2	MDA_C3	MDA_P1	MDA_P2	MDA_P3
hsa-miR-let-7a2	6.15724497	3.5829139	5.39292744	7.51267114	7.09831886	8.03176209
hsa-miR-21	7.15746323	7.1170116	7.21038404	8.04968396	7.73184759	7.59333543
hsa-miR-23b	5.34052884	5.2305848	5.34144343	6.41264785	6.23671298	6.10521338
hsa-miR-24-2	5.88820889	6.19117536	6.12006285	6.77731695	6.71089276	6.53246214
hsa-miR-27b	5.4192794	5.83370739	5.76341658	6.48587241	6.6264489	6.36897635
hsa-miR-29c	4.5735029	4.84285022	4.92474852	5.71135437	5.66552615	5.43786004
hsa-miR-30e	6.33810711	6.29430495	6.58309257	7.26305928	7.0724631	7.18565037
hsa-miR-32	4.91158514	4.13811278	5.28040154	6.27825208	6.42882328	6.06224558
hsa-miR-100	3.44343308	3.04302568	3.38254165	4.98075488	5.14505271	5.37249725
hsa-miR-181b1	5.45525519	5.47201061	5.41456543	7.52790433	7.46319394	7.31726317
hsa-miR-218-1	4.73144022	4.81238275	4.39950474	5.49569579	5.52324233	5.27959563
hsa-miR-221	6.46538236	5.22718267	6.12667247	7.9868455	7.7539962	7.58967958
hsa-miR-222	5.30497784	4.96074582	5.37758376	6.14011298	6.02963364	6.03950445
hsa-miR-301a	3.10465299	2.85008898	3.00888754	3.63334488	3.34090809	3.8967365
hsa-miR-421	6.14263361	4.97932177	5.67140845	7.23117174	6.81012158	7.43336182
hsa-miR-424	6.4033721	6.15607422	6.31082714	7.04718636	6.98788762	6.92718311
hsa-miR-454	3.16824989	3.08251009	3.0479751	3.90756546	3.86234534	3.42017826
hsa-miR-503	5.90153516	5.95386136	5.93516611	6.72664691	6.88801779	6.8197469
hsa-miR-505	3.22514005	3.09123411	3.47866881	5.1773951	4.25902727	3.7622065
hsa-miR-539	3.12428945	3.27656814	4.08263947	3.91586043	4.36224237	4.31800315
hsa-miR-548ak	2.00469954	2.1238871	1.93142606	2.54056814	3.23045928	2.32168539
hsa-miR-553	3.07376456	2.4468893	2.78425582	3.42876884	3.3306336	3.42236759
hsa-miR-570	6.03657026	5.5227412	6.1270458	7.04486101	6.88656421	6.9658515
hsa-miR-573	4.07893174	3.52672096	4.11196939	5.08361722	5.04749807	4.68288916
hsa-miR-579	5.61224293	4.95473986	5.48872818	5.98970896	5.8943887	6.24230841
hsa-miR-597	4.09376171	3.43442889	4.19627658	4.88980335	4.48915529	4.53288977
hsa-miR-604	6.07962989	4.86921135	6.0110496	7.02804111	7.09945008	6.75232412
hsa-miR-619	6.04732356	5.94627538	6.18481617	6.6144722	6.82910145	6.68924882
hsa-miR-626	2.76621771	2.34204257	2.64637308	3.43597061	3.29792697	3.18188037
hsa-miR-634	3.7646693	4.07526247	3.56336183	4.35993191	4.43583262	4.48119853
hsa-miR-646	6.01943376	6.2156874	6.35073819	7.12395719	6.87281164	6.66883954
hsa-miR-924	3.76333148	3.61987432	4.03229553	4.99446741	5.08653044	5.17132598
hsa-miR-1206	4.08572491	2.79680592	3.61261344	4.35929675	4.64478732	4.74269551

hsa-miR-1250	5.28037034	5.06858736	5.47059296	6.06326406	5.93519489	5.78861094
hsa-miR-1254-1	5.80722226	4.72419695	5.51023106	6.35435038	6.12266797	6.22304257
hsa-miR-1260b	3.165828	2.86052039	2.85348303	3.47307187	3.95144329	3.47282024
hsa-miR-1284	2.27571437	2.08786048	2.48620666	2.54038943	3.17618044	3.00601933
hsa-miR-1299	5.31933721	4.76123823	4.53702272	6.43021488	5.52398093	6.25790157
hsa-miR-3118-2	4.80145019	4.7626248	4.64036846	5.39232813	5.40169766	5.65292171
hsa-miR-3161	4.98520072	5.13868082	5.09725793	6.03447854	5.82823218	6.01709497
hsa-miR-3671	3.28967648	3.07979799	3.04206924	3.66764464	3.9766414	3.98249454
hsa-miR-3685	5.05261531	3.97036783	4.4161285	5.41066244	5.42352887	5.92347095
hsa-miR-3925	6.95842894	6.73475823	6.4016665	7.47855577	7.20445358	7.5031358
hsa-miR-3942	2.71412212	2.61908388	2.81380785	3.70511306	3.44792398	3.15743078
hsa-miR-4677	5.04407021	4.37213025	5.04714672	5.75658741	5.82057693	5.12261322
hsa-miR-4681	4.66272755	4.83133322	4.87450644	5.38705883	5.8546086	5.39025569
hsa-miR-5047	6.93959027	6.09714154	6.8708762	8.28275328	8.20565992	8.06359697
hsa-miR-4295	4.1270149	3.57226721	3.66117376	5.21726237	4.56108611	5.02350201
hsa-miR-4435-1	4.11453757	3.28795208	3.93806547	4.89686795	4.70044095	4.39851607
hsa-miR-4451	4.78164288	3.76606997	3.79335811	5.73447332	5.49908456	5.30768172
hsa-miR-4454	5.80349003	6.10969493	6.04639292	7.05399433	6.66776529	6.62982385
hsa-miR-4462	4.9861882	4.46164397	4.57027179	5.35444468	5.50462001	5.28771849
hsa-miR-4473	2.74001514	2.63535289	2.45925502	3.20374878	2.95004738	3.479499
hsa-miR-4500	2.06274153	2.3613557	2.42215842	5.78029765	5.45422647	6.09121084
hsa-miR-4504	4.77717181	4.30733159	4.80878027	5.57716846	5.14525546	5.43428494

RMA normalized probe intensity values of up-regulated miRNAs. Columns correspond to three control samples (MDA_C1 to C3) and three samples where Prrx1 was induced (MDA_P1 to P3)
