# Supplementary material for: A gene regulatory network to control EMT programs in development and disease
Source: Nat Commun. 2019 Nov 11;10:5115. doi: 10.1038/s41467-019-13091-8 (PMC6848104; doi:10.1038/s41467-019-13091-8)
Supplement: Supplementary file 4 — Reporting Summary [file 41467_2019_13091_MOESM4_ESM.pdf]

nature research

Corresponding author(s): M. Angela Nieto

Last updated by author(s): Oct 3, 2019

Reporting Summary

Nature Research wishes to improve the reproducibility of the work that we publish. This form provides structure for consistency and transparency in reporting. For further information on Nature Research policies, see Authors & Referees and the Editorial Policy Checklist.

Statistics

For all statistical analyses, confirm that the following items are present in the figure legend, table legend, main text, or Methods section.

n/a	Confirmed	
☐	☒	The exact sample size (n) for each experimental group/condition, given as a discrete number and unit of measurement
☐	☒	A statement on whether measurements were taken from distinct samples or whether the same sample was measured repeatedly
☐	☒	The statistical test(s) used AND whether they are one- or two-sided<br>*Only common tests should be described solely by name; describe more complex techniques in the Methods section.*
☒	☐	A description of all covariates tested
☐	☒	A description of any assumptions or corrections, such as tests of normality and adjustment for multiple comparisons
☐	☒	A full description of the statistical parameters including central tendency (e.g. means) or other basic estimates (e.g. regression coefficient) AND variation (e.g. standard deviation) or associated estimates of uncertainty (e.g. confidence intervals)
☐	☒	For null hypothesis testing, the test statistic (e.g. F, t, r) with confidence intervals, effect sizes, degrees of freedom and P value noted<br>*Give P values as exact values whenever suitable.*
☒	☐	For Bayesian analysis, information on the choice of priors and Markov chain Monte Carlo settings
☒	☐	For hierarchical and complex designs, identification of the appropriate level for tests and full reporting of outcomes
☒	☐	Estimates of effect sizes (e.g. Cohen's d, Pearson's r), indicating how they were calculated

Our web collection on statistics for biologists contains articles on many of the points above.

Software and code

Policy information about availability of computer code

Data collection	No new software was used.
Data analysis	Data analysis was performed with Microsoft Excel 2013, GraphPad Prism 8.0.1, ImageJ Fiji version 1.52i. Images were prepared using Adobe Photoshop and Adobe Illustrator CS6.

For manuscripts utilizing custom algorithms or software that are central to the research but not yet described in published literature, software must be made available to editors/reviewers. We strongly encourage code deposition in a community repository (e.g. GitHub). See the Nature Research guidelines for submitting code & software for further information.

Data

Policy information about availability of data

All manuscripts must include a data availability statement. This statement should provide the following information, where applicable:

- Accession codes, unique identifiers, or web links for publicly available datasets
- A list of figures that have associated raw data
- A description of any restrictions on data availability

The microarray data generated in this study, as well as public scRNA-seq data datasets analyzed during the current study are available in the Gene Expression Omnibus (GEO) [https://www.ncbi.nlm.nih.gov/geo/] repository under the following accession numbers that are also listed in Supplementary table 1. Microarray in MDA231 cells: GSE138078 [https://www.ncbi.nlm.nih.gov/geo/query/acc.cgi?acc=GSE138078], Zebrafish embryos: GSM3067194 [https://www.ncbi.nlm.nih.gov/geo/query/acc.cgi?acc=%20GSM3067194], mouse embryo: GSE87038 [https://www.ncbi.nlm.nih.gov/geo/query/acc.cgi?acc=GSE87038], head and neck cancer patients: GSE103322 [https://www.ncbi.nlm.nih.gov/geo/query/acc.cgi?acc=GSE103322] and breast cancer patients: GSE75688 [https://www.ncbi.nlm.nih.gov/geo/query/acc.cgi?acc=GSE75688]. The source data underlying Figs 1c, f-h, 2a–f, 3b-f, 4a-e and h, and 6a, and Supplementary Figs 1f-g, 2a-f, 3a and c-i, 4a and g-j, 5c and e, and 7b are provided as a Source Data file.

Field-specific reporting

Please select the one below that is the best fit for your research. If you are not sure, read the appropriate sections before making your selection.

☒ Life sciences ☐ Behavioural & social sciences ☐ Ecological, evolutionary & environmental sciences

For a reference copy of the document with all sections, see nature.com/documents/nr-reporting-summary-flat.pdf

Life sciences study design

All studies must disclose on these points even when the disclosure is negative.

Sample size

For breast cancer patients' overall survival analyses: total n=1402, lymph-node positive patients n=313 and basal subtype n=241 (Fig. 5 and Supp. 6)

For breast and head-and-neck cancers single cell RNA-seq datasets: total n=764 (after exclusion n= 440) and n=6962 (after exclusion n= 1620), respectively (Fig. 1g-h)

For mouse and zebrafish embryos single cell RNA-seq datasets: total n=551 (after exclusion n= 138) and n=5902 (after exclusion n= 1718), respectively (Fig. 1c and f)

For snail1a/b expression pattern analysis in zebrafish embryos, sample size was estimated using GPower 3.1 where values were set at p = 0.05 and beta = 0.8. Control n=30 and Sponge injected = 96 (Fig. 4f)

In mouse embryo mutant for Prrx1, change in Snail1 expression pattern was analyzed by IF and confirmed in 2 mutant embryos compared to 3 WT (Fig. 4i and Supp. 5e). Western blot was performed for WT n=5 and mut n=4 embryos (Fig. 4h and Supp. 5b)

For snail1a/b, prrx1a/b and ptrmiRs qPCR in zebrafish embryos treated with water (control) or BMP (Fig. 6a) each independent experiment include pools of 6 randomly chosen embryos.

For SNAIL1 and PRRX1 ISH in chicken embryos treated with control or BMP beads (Fig. 6b): for control (14/14 for both SNAIL1 and PRRX1), BMP 1h (SNAIL1 8/8, PRRX1 6/6), BMP 5h (SNAIL1 10/11, PRRX1 14/16) and BMP 10h (SNAIL1 5/6, PRRX1 5/5)

For SNAIL1 and PRRX1 IF in chicken embryos treated with control or BMP beads (Supp Fig. 7a) 6-8 somites from 2 embryos per condition, per time point were analyzed.

For expression analyses via ISH at least in 2 embryos per condition were analyzed. Detail as follows:

Mouse embryos, E8.5 n=5, E9.5 n=5 (Fig. 1e and Supp. 1b-d)

zebrafish embryos: n=3 (Fig. 1a), double ISH n=3 (fig1b)

chicken embryos: n=2 (Fig. 1d)

miRISH mouse: n=3 (Fig. 3g)

miRISH zebrafish n=2 (Fig. 4g)

Double IF mouse embryos: n= 2 (Fig. 6b), n=3 (Supp. 8b)

For measuring the average nuclei signal intensity for double IF in three different cell lines (Supp. 1f) at least 5 different ransom plains were taken: SUM149PT n=1389, MDA436 n= 1872 and BT549 n= 899.

For measuring the average nuclei signal intensity for IF in mouse embryo sections: (Supp. 5d) WT n=401 and mut n=410, and (Supp. 5f) WT n=1742 and mut n=1858.

For luciferase assays and qPCR sample sizes were not calculated, but standard repetition of experiments was followed to estimate a normal distribution of the data.

Data exclusions

In the analyses of single cell RNA-seq datasets, the cells with no value for both Snail1 and Prrx1 were excluded.

Replication

All replication attempts of the experiments were successful.

Randomization

All animals were randomly selected for experiments.

Blinding

At least two individuals checked the analysis of experiments.

Reporting for specific materials, systems and methods

We require information from authors about some types of materials, experimental systems and methods used in many studies. Here, indicate whether each material, system or method listed is relevant to your study. If you are not sure if a list item applies to your research, read the appropriate section before selecting a response.

Materials & experimental systems

n/a	Involved in the study
☐	☒ Antibodies
☐	☒ Eukaryotic cell lines
☒	☐ Palaeontology
☐	☒ Animals and other organisms
☒	☐ Human research participants
☐	☒ Clinical data

Methods

n/a	Involved in the study
☒	☐ ChIP-seq
☒	☐ Flow cytometry
☒	☐ MRI-based neuroimaging

Antibodies

Antibodies used

Prrx1 (for ChIP): Sigma, Rabbit polyclonal (HPA051084)
IgG: Diagenode, Rabbit (C15410206)
Myc tag: Abcam, goat pAb (ab9132)
Prrx1 (for IF): Tanaka lab
Snail1 (for IF in embryo): Cell Signaling, rabbit monoclonal (C15D3)
Snail1 (for IF in cultured cells): Cell Signaling, rat monoclonal (SN9H2)
GFP: Chicken polyclonal, Aveslab (2BScientific), GFP-1020
DIG-AP Fab fragments: sheep polyclonal, Roche (11093274910)
DIG-POD Fab fragments: sheep polyclonal, Roche (11207733910)
FLUO-POD Fab fragments: sheep polyclonal, Roche (11426346910)
beta-actin: Rabbit polyclonal, Abcam
Alexa Fluor 488, goat anti-rabbit Invitrogen (A11008)
Alexa Fluor 568, goat anti-rabbit Invitrogen (A11011)
Alexa Fluor 488, goat anti-chicken Life technologies (A11039)
Alexa Fluor 568, goat anti-rat Invitrogen (A11077)
Snail1 (for IF in cells), Rat monoclonal Cell signaling (SN9H2, #4719)
Histon H3, Rabbit polyclonal Abcam (ab1791)

Validation

All validations were performed before in Ocana et al. 2017, Nature.

Eukaryotic cell lines

Policy information about cell lines

Cell line source(s)

ATCC and ASTERAND BIOSCIENCE

Authentication

Cell lines were authenticated using STR profile by the Genetic Analysis Service at Miguel Hernandez University, Spain.

Mycoplasma contamination

All cell lines were tested and confirmed negative for mycoplasma on a monthly bases at the host institution.

Commonly misidentified lines
(See ICLAC register)

No commonly misidentified lines were used.

Animals and other organisms

Policy information about studies involving animals; ARRIVE guidelines recommended for reporting animal research

Laboratory animals

Fertilized eggs from zebrafish strain AB, fertilized hen eggs, and mouse embryos strain C57BL/6J or FVB/NJ were used.
All zebrafish embryos were between 12 and 24 hours post fertilization.
All chicken embryos were between 26-30 hours post fertilization.
All mouse embryos were 8.5, 9.5 or 11.5 days post coitum.

Wild animals

Study did not involve use of wild animals.

Field-collected samples

Study did not involve samples collected from the field.

Ethics oversight

We affirm to have complied with all relevant ethical regulations for animal testing and research as follows. All animal procedures were conducted in compliance with the European Community Council Directive (2010/63/EU) and Spanish legislation. The

protocols were approved by the CSIC Ethical Committee and the Animal Welfare Committee at the Institute of Neurosciences, Alicante

Note that full information on the approval of the study protocol must also be provided in the manuscript.

Clinical data

Policy information about clinical studies

All manuscripts should comply with the ICMJE guidelines for publication of clinical research and a completed CONSORT checklist must be included with all submissions.

Clinical trial registration	The study did not include any clinical trial registration.
Study protocol	Not applicable.
Data collection	Clinical data including survival analyses for breast cancer patients were obtained from public database: http://kmplot.com/analysis. Single cell RNA sequencing data from breast and head-and-neck cancre patients were obtained from GEO database, with references GSE75688 and GSE103322, respectively.
Outcomes	Not applicable.
